# Use of Continuous Glucose Monitoring in Non-diabetic Individuals for Cardiovascular Prevention: A Systematic Review of Its Impact on Guiding Lifestyle Interventions

**DOI:** 10.7759/cureus.94460

**Published:** 2025-10-13

**Authors:** Nour Ahmed, Mohamed Faisal Elzein Ali, Muhanad Noureldin Hamed Mohamed, Rayan Saad Aldeen Mohammed Saad Aldeen, Rayan Ismat Ibrahim Omer, Tasabeeh Musa Adam Omer, Riham Abdelmagid ElTahir Hamza, Malak Rabih Musa Rabih

**Affiliations:** 1 Endocrine and Diabetes, George Eliot Hospital National Health Service (NHS) Trust, Nuneaton, GBR; 2 Medicine, National Guard Health Affairs (NGHA), Madina, SAU; 3 Police Health Services, Saad Abdullah Academy Hospital, Kuwait City, KWT; 4 Medicine, Barking, Havering and Redbridge University Hospitals National Health Service (NHS) Trust, London, GBR; 5 Medicine, Igraa College of Science and Technology, Wad Madani, SDN; 6 Stroke Medicine, Barts Health National Health Service (NHS) Trust, London, GBR; 7 Family Medicine, Nad Al Hamar Health Center, Dubai, ARE; 8 Internal Medicine, King Salman Armed Forces Hospital, Tabuk, SAU; 9 Internal Medicine, National University Sudan, Nottingham, GBR

**Keywords:** cardiovascular prevention, continuous glucose monitoring, glycemic variability, lifestyle intervention, non-diabetic, physical activity, systematic review

## Abstract

Cardiovascular disease (CVD) is a leading global cause of mortality, with prevention through lifestyle modification being paramount. Continuous glucose monitoring (CGM), which provides real-time, dynamic glycemic data, is increasingly being explored in non-diabetic populations to guide lifestyle interventions. This systematic review aims to synthesize the evidence on the use of CGM in non-diabetic individuals for guiding lifestyle modifications and assess its impact on cardiovascular risk prevention. A systematic literature search was conducted in PubMed/MEDLINE, Embase, Scopus, Web of Science, and ClinicalTrials.gov from January 2020 to August 2025. Studies investigating CGM-guided lifestyle interventions in non-diabetic adults and reporting cardiovascular risk factors or metabolic outcomes were included. Study selection, data extraction, and risk of bias assessment (using Cochrane Risk of Bias 2 (RoB 2) for randomized controlled trials (RCTs) and Risk Of Bias In Non-randomized Studies of Interventions (ROBINS-I) for non-randomized studies) were performed by independent reviewers. A narrative synthesis was conducted due to study heterogeneity. Seven studies were included. CGM was used to personalize exercise timing, such as initiating walking before an individual's postprandial glucose peak, which significantly reduced postprandial glucose, insulin, and C-peptide levels. CGM also served as a motivational tool, increasing readiness for physical activity. Observational studies demonstrated CGM's ability to identify subclinical dysregulation in conditions like menopause and obstructive sleep apnea, linking higher glycemic variability to surrogate markers of cardiovascular risk, such as blood pressure variability. The overall risk of bias was low for six studies and serious for one, primarily due to confounding. Direct evidence on changes in traditional cardiovascular risk factors was limited. CGM shows promise for personalizing lifestyle interventions and improving glycemic outcomes in non-diabetic individuals, which are key surrogates for cardiovascular risk. Its utility lies in optimizing physical activity timing, enhancing motivation, and identifying at-risk metabolic phenotypes. However, evidence of a direct impact on hard cardiovascular endpoints remains limited. Future long-term trials should focus not on reaffirming the established link between glycemic variability and cardiovascular risk but on evaluating whether CGM-guided behavioral interventions offer a cost-effective and sustainable approach to enhancing cardiovascular prevention strategies in non-diabetic populations.

## Introduction and background

Cardiovascular disease (CVD) remains the leading cause of morbidity and mortality worldwide, accounting for nearly one-third of all global deaths [1]. Despite advances in pharmacological therapies and interventional strategies, prevention through lifestyle modification continues to be the cornerstone of reducing cardiovascular risk [2]. Traditional risk stratification methods rely on clinical parameters such as blood pressure, lipid profiles, and body mass index (BMI), yet these markers may not fully capture the dynamic interplay between metabolic health and cardiovascular outcomes. Increasingly, attention has turned to the role of glycemic variability and subclinical dysglycemia-even among individuals without a diagnosis of diabetes-as potential contributors to cardiovascular risk [3].

Continuous glucose monitoring (CGM) has transformed the management of diabetes by providing real-time, ambulatory glucose measurements that capture fluctuations invisible to conventional point-in-time testing [4]. In recent years, CGM has also been adopted in research and clinical practice involving non-diabetic populations [5]. Evidence suggests that glucose excursions within the normoglycemic range may influence vascular function, inflammation, and cardiometabolic health [6]. By providing granular data on glucose trends, CGM offers a unique opportunity to personalize lifestyle interventions such as diet, physical activity, and sleep patterns, thereby potentially mitigating cardiovascular risk before overt metabolic disease develops [7].

Several observational and interventional studies have begun to explore the application of CGM in non-diabetic individuals for lifestyle optimization and cardiovascular prevention. However, the evidence is fragmented, with studies differing in populations, intervention strategies, outcome measures, and duration of follow-up. Moreover, while CGM has demonstrated promise in improving patient engagement and behavior change, its overall impact on guiding lifestyle interventions specifically aimed at cardiovascular risk reduction has not been systematically evaluated [8].

The present systematic review aims to synthesize the available evidence on the use of CGM in non-diabetic individuals, with a particular focus on its role in informing lifestyle interventions for cardiovascular prevention. By critically appraising the current literature, we seek to clarify the potential utility of CGM as a preventive tool, identify gaps in knowledge, and highlight implications for future clinical practice and research.

## Review

Methodology

Protocol and Reporting

This systematic review was conducted in accordance with the Preferred Reporting Items for Systematic Reviews and Meta-Analyses (PRISMA) 2020 checklist to ensure transparency, reproducibility, and methodological rigor [9]. All stages of the review process, including study selection, data extraction, and risk of bias assessment, followed predefined eligibility criteria and review protocols.

Eligibility Criteria

We included studies published between January 2020 and August 2025, as our aim was to capture the most recent and relevant evidence on the application of CGM in non-diabetic populations for cardiovascular prevention and lifestyle modification. Eligible studies included randomized controlled trials (RCTs), non-randomized interventional studies, and observational studies that investigated the use of CGM to guide lifestyle interventions (such as diet, exercise, or behavioral change) in individuals without a diagnosis of diabetes mellitus. Studies were required to report outcomes related to cardiovascular risk factors (e.g., blood pressure, lipid profiles, body weight, physical activity, or dietary modification), metabolic markers, or patient engagement. Publications not in English, conference abstracts without full texts, reviews, editorials, and case reports were excluded.

Information Sources and Search Strategy

A comprehensive literature search was conducted in five electronic databases: PubMed/MEDLINE, Embase (Elsevier), Scopus, Web of Science, and ClinicalTrials.gov. The search period covered January 2020 to August 2025, with the last search performed on August 30, 2025. The search strategy was designed in consultation with an experienced medical librarian and used a combination of controlled vocabulary terms (e.g., MeSH) and free-text keywords related to “continuous glucose monitoring,” “non-diabetic,” “cardiovascular prevention,” and “lifestyle interventions.” Reference lists of included studies and relevant reviews were also screened manually to identify additional eligible articles.

Study Selection

All identified records were imported into EndNote X9 (Clarivate Analytics, London, UK), where duplicates were automatically removed and then manually verified for accuracy. Title and abstract screening was independently performed by two reviewers, followed by full-text review to determine final eligibility. Any disagreements between reviewers were resolved through discussion or consultation with a third reviewer.

Data Extraction

Data were extracted into a standardized form developed by the review team. Extracted information included study characteristics (author, year, country, design, and sample size), population demographics, type and duration of CGM intervention, comparator(s), outcomes assessed, and key findings related to lifestyle modification and cardiovascular prevention. Data extraction was performed by one reviewer and independently verified by a second reviewer to ensure accuracy.

Risk of Bias Assessment

The quality and risk of bias of included studies were assessed using validated tools appropriate for study design. For RCTs, the Cochrane Risk of Bias 2 (RoB 2) tool was employed [10], while the Risk Of Bias In Non-randomized Studies of Interventions (ROBINS-I) tool was used for non-randomized and observational studies [11]. Each study was evaluated across multiple domains, and judgments were classified as low risk, some concerns, or high risk of bias. Assessments were conducted independently by two reviewers, with discrepancies resolved by consensus.

Data Synthesis

Given the heterogeneity across studies in terms of population characteristics, intervention strategies, outcome measures, and follow-up duration, a quantitative synthesis (meta-analysis) was not feasible. The included studies varied widely in their methodological approaches and endpoints, which precluded the pooling of data in a statistically meaningful way. Instead, findings were synthesized narratively, with particular attention to the consistency of evidence, emerging trends, and implications for cardiovascular prevention in non-diabetic individuals.

Ethical Considerations

This study is a systematic review based on previously published research. No new human or animal participants were involved; therefore, ethical approval was not required.

Results

Study Selection Process

**Figure 1:**
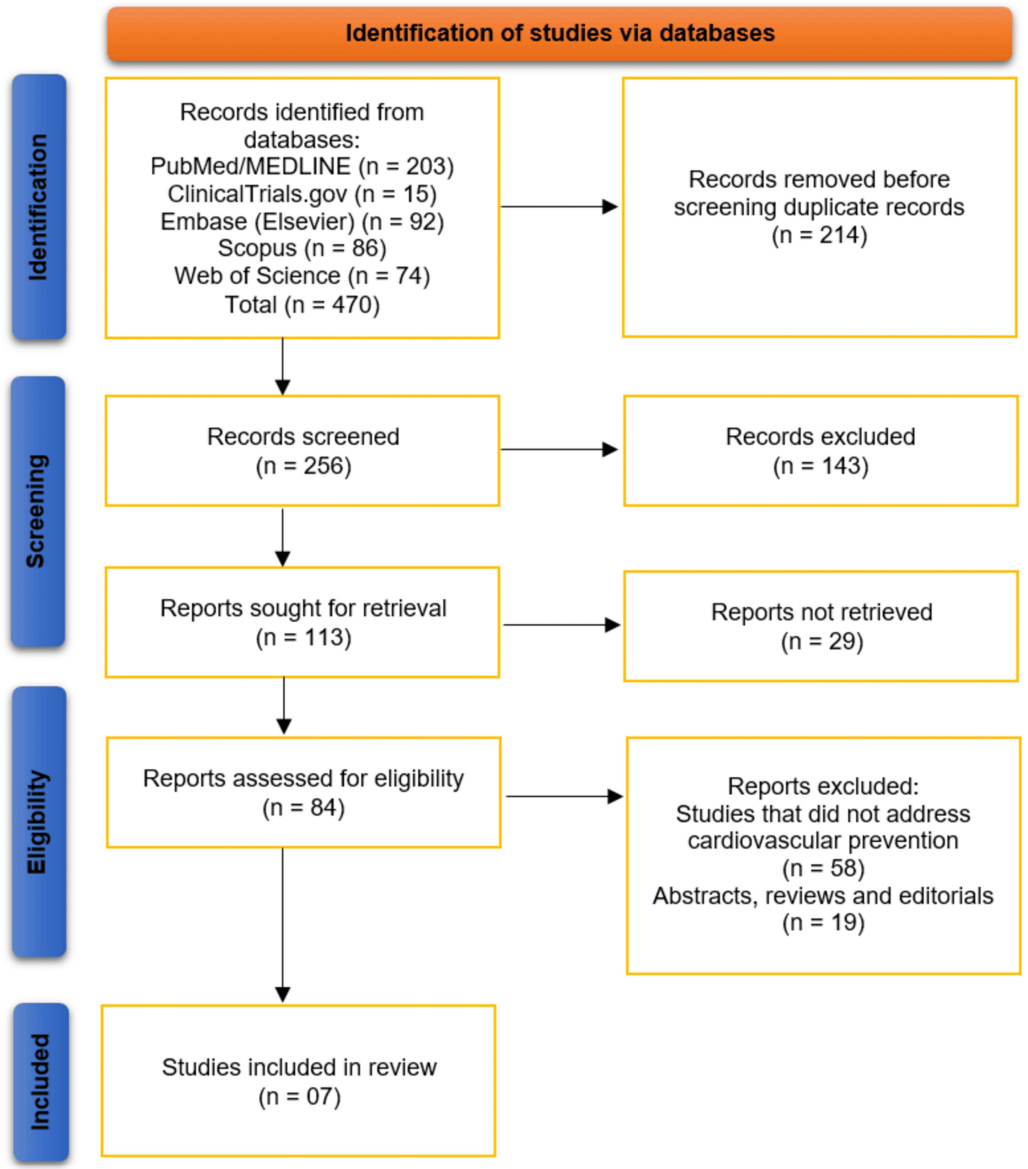
Studies Selection Process on PRISMA Diagram This figure illustrates the Preferred Reporting Items for Systematic Reviews and Meta-Analyses (PRISMA) flow of information through the systematic review process. A total of 470 records were identified from five databases (PubMed/MEDLINE, ClinicalTrials.gov, Embase, Scopus, and Web of Science). After removing 214 duplicates, 256 unique records were screened, leading to the exclusion of 143 records. Of the 113 full texts sought, 29 were not retrievable. After eligibility assessment, 77 studies were excluded (58 did not address cardiovascular prevention and 19 were abstracts/reviews/editorials). Seven studies met the inclusion criteria and were included in the final review [12-18].

The systematic search across five databases and registers (PubMed/MEDLINE, ClinicalTrials.gov, Embase, Scopus, and Web of Science) initially identified 470 records. After the removal of 214 duplicate records, 256 unique titles and abstracts were screened for relevance. This screening process led to the exclusion of 143 records, resulting in 113 reports being sought for full-text retrieval. Of these, 29 reports could not be retrieved. The remaining 84 full-text articles were assessed for eligibility against the review's inclusion criteria. This assessment resulted in the exclusion of 58 studies that did not address cardiovascular prevention and 19 articles that were abstracts, reviews, or editorials. Consequently, a total of seven studies met all eligibility criteria and were included in the qualitative synthesis of this systematic review [12-18]. The selection process is summarized in the PRISMA flow diagram (Figure 1).

Studies' Characteristics

The key characteristics of these studies are summarized in Table 1. The included studies were published between 2020 and 2023 and were conducted in a diverse range of countries, including China, France, Sweden, Turkey, the United States, South Korea, and the United Kingdom [12-18]. Study designs varied, encompassing three RCTs [12,14], one pilot intervention study [16], and three observational studies (one experimental bed rest model [13], one cross-sectional [15,17], and one large cohort study [18]). The total sample size across studies was approximately 1,127 non-diabetic participants, with individual study sizes ranging from N = 16 to N = 1,002. The populations studied were heterogeneous, including individuals who were overweight or obese [12,14,16], healthy men in a controlled bed rest model [13], normoglycemic normotensive adults [15], patients with obstructive sleep apnea (OSA) [17], and a large female cohort stratified by menopausal status [18]. The duration of CGM use also varied, from a single night [17] and short-term periods of 48 hours to 10 days [15,16] up to several weeks [14] or specific phases of an intervention [13]. The comparator groups included passive controls (e.g., habitual lifestyle [14]), within-subject comparisons (e.g., pre- vs. post-intervention [13]), or subgroup analyses (e.g., by menopausal status [18] or OSA severity [17]). Primary outcomes consistently focused on glycemic parameters, including postprandial glucose, glycemic variability, and time in range, with several studies also assessing cardiovascular risk surrogates such as blood pressure, lipids, and body composition [12,15,18].

**Table 1:** Key Characteristics of Included Studies This table summarizes the design, population, intervention, comparator, and primary outcomes of the seven included studies assessing CGM in non-diabetic individuals for cardiovascular prevention. Study designs comprised RCTs [12,14], an experimental bed rest model [13], observational cross-sectional studies [15,17], a pilot intervention study [16], and a large cohort study [18]. The populations included overweight/obese adults, healthy males, patients with OSA, and women across menopausal stages. CGM was applied for varying durations (single night to several weeks) and was used to guide or monitor lifestyle interventions. Primary outcomes included PPG, glycemic variability, insulin, C-peptide, and surrogate cardiovascular risk markers. CGM: continuous glucose monitoring; RCT: randomized controlled trial; PPG: postprandial glucose; BMI: body mass index; CGMS: continuous glucose monitoring system; tAUC: total area under the curve; HIEC: hyperinsulinemic-euglycemic clamp; MAG: mean absolute glucose; OGTT: oral glucose tolerance test; MBG: mean blood glucose; MAGE: mean amplitude of glycemic excursion; MODD: mean of daily differences; CV: coefficient of variation; BP: blood pressure; SBP: systolic blood pressure; PA: physical activity; OSA: obstructive sleep apnea

Author, year	Country	Study design	Population (non-diabetic status, N, age, sex)	CGM type/duration	Comparator	Follow-up duration	Primary outcomes assessed
Zhang et al. [12] (2021)	China	RCT	Overweight/obese, non-diabetic men; N = 20; age: 23.0 ± 4.3 years; sex: male	CGM used to predetermine individual PPG peak time (duration not explicitly stated)	Sitting (SIT) vs. walking at PPG peak (iP) vs. walking 20 min before PPG peak (20iP)	Each trial lasted 240 min; washout 6–14 days between conditions	PPG, plasma insulin, C-peptide concentrations
Trim et al. [13] (2023)	France	Experimental study (bed rest model)	Non-diabetic, 20 healthy males, age: 34 ± 8 years, BMI: 23.5 ± 1.8 kg/m²	CGMS, 5 days pre- and 5 days toward end of 60-day bed rest	Pre-bed rest vs. post-bed rest (within-subject comparison)	60 days (bed rest intervention)	Daily glycemia (interstitial glucose concentrations, tAUC), fasting glucose & insulin, whole-body glucose disposal (HIEC), glycemic variability (SD, J-index, M-value, MAG)
Smith et al. [14] (2021)	Sweden	RCT	Non-diabetic adults with obesity; N = 16; median age 50 (IQR 44–53) years; 10 women, 6 men	Continuous glucose monitor for 4 weeks	Habitual lifestyle (control)	4 weeks	Glycemic control (fasting glucose, daily glucose variation, OGTT, 24-h glucose curves), activity behavior, clinical chemistry, skeletal muscle lipidome
Sezer et al. [15] (2021)	Turkey	Observational (cross-sectional)	Normoglycemic, normotensive individuals, N = 27, age 23.8 ± 2.7 years, 66% women	CGM, 48 hours	None	48 hours	Glycemic variability parameters (MBG, SD, MAGE, MODD, CV, daytime/nighttime glucose) and correlation with BP variability (SD of 24-h SBP, SD of daytime SBP)
Liao et al. [16] (2020)	USA	Pilot intervention	Non-diabetic overweight/obese adults, N = 19, age not specified, 84% female	CGM, 10 days	Fitbit tracker	10 days	Acceptability of CGM and PA education module, changes in motivational readiness for PA
Byun et al. [17] (2020)	Republic of South Korea	Observational, cross-sectional	Non-diabetic, N = 23 (11 moderate-severe OSA, 12 no/mild OSA)	CGM every 5 min during overnight polysomnography	No/mild OSA vs. moderate-severe OSA	Single night	Nocturnal glucose changes; relationship with OSA features (sympathetic hyperactivation, intermittent hypoxemia, sleep fragmentation)
Bermingham et al. [18] (2022)	UK	Observational cohort (PREDICT 1)	Non-diabetic females; n = 1,002 (pre-menopause n = 366, peri-menopause n = 55, post-menopause n = 206); age not specified; sex: female	CGM; 0–6 h postprandial measurements	Age-matched subgroup comparisons; pre- vs. post-menopause	Cross-sectional (single visit; postprandial measurements over 6 h)	Fasting and PPG and insulin responses, glycemic variability, time in range, HbA1c, GlycA (inflammation), diet, sleep, visceral fat, triglycerides, gut microbiome

Impact of CGM-Guided Lifestyle Interventions on Glycemic Outcomes

The findings from the included studies demonstrate that CGM can be effectively used to guide and evaluate lifestyle interventions, leading to improvements in glycemic control in non-diabetic populations. The main findings are detailed in Table 2.

**Table 2:** Main Findings of Included Studies This table presents the key outcomes from CGM-guided lifestyle interventions or observational use in non-diabetic populations. Interventions ranged from exercise timed to PPG peaks [12], reduced energy intake during inactivity [13], activity breaks prompted by wearable devices [14], and motivational use of CGM feedback [16]. Observational studies explored associations between glycemic variability and BP [15], nocturnal glucose changes in OSA [17], and postprandial responses across menopausal status [18]. Reported outcomes included reductions in PPG, insulin, and C-peptide [12], improved fasting glucose and glycemic variability [14], correlations between glycemic and BP variability [15], and heightened cardiovascular risk profiles in post-menopausal women [18]. CGM: continuous glucose monitoring; BP: blood pressure; BMI: body mass index; PPGP: postprandial glucose peak; AUC: area under the curve; PPG: postprandial glucose; HIEC: hyperinsulinemic-euglycemic clamp; tAUC: total area under the curve; MAG: mean absolute glucose; CV: coefficient of variation; OGTT: oral glucose tolerance test; LDL: low-density lipoprotein; MBG: mean blood glucose; MODD: mean of daily differences; MAGE: mean amplitude of glycemic excursions; PA: physical activity; OSA: obstructive sleep apnea; MHT: menopausal hormone therapy

Author, year	Lifestyle intervention guided by CGM (e.g., diet, exercise, behavior change)	Key findings on lifestyle modification (adherence, changes in diet/exercise, weight loss, etc.)	Glycemic outcomes (time in range, glucose variability, mean glucose)	Cardiovascular risk outcomes (BP, lipids, BMI, surrogate markers)
Zhang et al. [12] (2021)	Exercise (30 min walking at 50% VO₂max) timed based on individualized CGM-determined PPGP	Participants adhered to CGM-guided walking protocols (sitting, walking at PPGP, walking 20 min before PPGP). Exercise timing influenced effectiveness, with pre-PPGP walking being superior	Walking 20 min before PPGP reduced 4-h incremental AUC for plasma PPG (−0.6 mmol·L⁻¹·h; p = 0.047), insulin (−28.7%, p < 0.001), and C-peptide (−28.7%, p < 0.001). Walking at PPGP also lowered C-peptide (−14.9%, p = 0.001). Effects were strongest in men with BMI > 27.5 kg/m²	Reduction in BMI-related subgroup, but no direct measures of BP or lipids reported. Surrogate cardiometabolic benefits observed via lower insulin and C-peptide levels
Trim et al. [13] (2023)	Energy intake reduction during prolonged physical inactivity (bed rest); diet adjusted to match reduced energy expenditure while meal composition and timing maintained	Caloric intake reduced by 20% and carbohydrate intake reduced by 25% to maintain energy balance during inactivity	Fasting plasma insulin ↑40%; glucose disposal ↓24% (via HIEC); daily glucose tAUC ↑6%; nocturnal glucose tAUC ↑9%; glycemic variability (SD, J-index, M-value, MAG) unchanged	BMI stable (energy balance maintained)
Smith et al. [14] (2021)	Frequent activity breaks from sitting (FABS) guided by smartwatch notifications (3 min low-to-moderate physical activity every 30 min between 08:00 and 18:00)	Modest increase in daily steps (+744 steps/day) and walking time (+10.4 min/day). Other activity/sedentary behavior indices largely unchanged. Adherence appears acceptable under free-living conditions	Fasting glucose decreased by −0.34 ± 0.37 mmol/L; daily glucose variability (% CV) decreased by −2% ± 2.2%; OGTT and 24-h average glucose curves unaffected	Minimal changes: tendency for decreased fasting LDL cholesterol; skeletal muscle lipidome largely unaltered; no significant changes in other clinical chemistry or BMI reported
Sezer et al. [15] (2021)	Study was observational, focusing on measuring glycemic and BP variability rather than guiding lifestyle interventions	Not reported	MBG, SD of blood glucose, MAGE, MODD, coefficient of variation, daytime and nighttime glucose measured; correlations with BP variability observed	Blood pressure variability (SD of 24-h SBP, SD of daytime SBP) correlated with glycemic variability (SD MBG, MAGE, MODD)
Liao et al. [16] (2020)	Physical activity education with one-on-one counseling + 10-day self-monitoring using CGM and Fitbit	High acceptability of counseling and monitoring; increased motivation for PA; significant movement from precontemplation to action stage in PA readiness	Acute effects of PA on glucose patterns highlighted to participants	No changes in BP, lipids, or other CV markers reported
Byun et al. [17] (2020)	None (observational)	Not reported	Moderate–severe OSA: glucose ↑ after sleep onset; mild/no OSA: glucose ↓; strong coupling with OSA features	Minimum oxygen saturation lower in moderate–severe OSA
Bermingham et al. [18] (2022)	Diet and behavior changes (dietary intake, sleep, potentially guided by CGM patterns)	Post-menopausal females had higher sugar intakes (+12%) and poorer sleep (-12%) compared with pre-menopausal females; diet and gut microbiome partially mediated associations with metabolic health	Postprandial glucose AUC (0–2 h peak +4%), 2 h glucose AUC (+42%), insulin AUC (+4%); CGM measures showed higher glycemic variability and reduced time in range post-menopause	Higher fasting glucose, HbA1c, inflammation (GlycA), and unfavorable postprandial triglycerides; MHT associated with improved visceral fat, fasting glucose/insulin, and postprandial triglycerides. Surrogate markers suggest increased cardiovascular risk post-menopause

In the context of physical activity, CGM was used to personalize the timing of exercise for maximal benefit. Zhang et al. [12] utilized CGM to determine the individual postprandial glucose peak (PPGP) time in overweight/obese men and found that initiating a 30-minute walk 20 minutes before this peak was superior to walking at the peak or sitting, resulting in significant reductions in four-hour incremental area under the curve (AUC) for plasma glucose, insulin, and C-peptide. This suggests that CGM can guide precise exercise timing to blunt postprandial glycemic excursions. Similarly, Smith et al. [14] used smartwatch notifications prompted by CGM patterns to encourage frequent activity breaks from sitting in free-living adults with obesity. While this intervention did not affect overall glucose tolerance, it successfully reduced fasting glucose and daily glycemic variability. Conversely, a study on enforced physical inactivity by Trim et al. [13] highlighted the negative glycemic impact of inactivity even when energy balance was maintained. Their bed rest model showed a significant increase in fasting insulin and a decrease in whole-body glucose disposal, underscoring the critical role of physical activity-monitored via CGM-in maintaining glucose homeostasis.

CGM also proved valuable as a motivational tool. Liao et al. [16] reported that a 10-day period of self-monitoring with CGM and a Fitbit significantly increased participants' motivation and readiness for physical activity, moving them from precontemplation to action stages. Although not designed to measure long-term glycemic changes, this pilot study demonstrated the high acceptability of using real-time CGM data to motivate behavioral change.

Beyond exercise, CGM data revealed important insights into other physiological states. Bermingham et al. [18] used CGM in the large PREDICT 1 cohort to show that post-menopausal women exhibited higher postprandial glucose AUC, greater glycemic variability, and reduced time in range compared to pre-menopausal women, differences that were partially mediated by diet and gut microbiome. This indicates CGM's utility in identifying populations at higher metabolic risk. Furthermore, Byun et al. [17] used CGM during polysomnography to reveal dynamic nocturnal glucose changes associated with OSA severity, linking sleep-related features like intermittent hypoxemia to acute glucose dysregulation.

Impact on Cardiovascular Risk Factors and Surrogate Markers

The evidence regarding the direct impact of CGM-guided interventions on traditional cardiovascular risk factors is more limited but suggestive of potential benefits. Several studies reported on surrogate markers. Zhang et al. [12] observed that the reduction in insulin and C-peptide following timed walking points to improved insulin sensitivity, a key cardiometabolic benefit, particularly in individuals with a higher BMI. The observational study by Sezer et al. [15] provided a direct link between glucose variability and cardiovascular risk by demonstrating a significant correlation between glycemic variability parameters (such as mean blood glucose, mean amplitude of glycemic excursions (MAGE), and mean of daily differences (MODD)) and blood pressure variability in normotensive individuals.

The study by Bermingham et al. [18] offered the most comprehensive assessment of cardiovascular risk, associating the poorer glycemic profile in post-menopausal women with higher fasting glucose, HbA1c, inflammation (GlycA), and unfavorable postprandial triglyceride levels. These findings suggest an increased cardiovascular risk profile post-menopause that can be effectively captured by CGM. In contrast, interventions like the frequent activity breaks study by Smith et al. [14] and the physical activity pilot by Liao et al. [16] did not report significant changes in blood pressure or lipid profiles, likely due to their short duration or specific focus. Similarly, the bed rest study by Trim et al. [13] maintained a stable BMI by design and did not report on other cardiovascular markers, focusing instead on glucoregulatory physiology.

Risk of Bias Assessment

The assessment of methodological quality revealed that the majority of included studies had a low risk of bias. Specifically, the two RCTs by Zhang et al. [12] and Smith et al. [14] were judged to have a low risk of bias across all domains of the Cochrane RoB 2 tool, including the randomization process, deviations from intended interventions, missing outcome data, measurement of outcomes, and selection of reported results. Among the non-randomized studies assessed with the ROBINS-I tool, the experimental study by Trim et al. [13], the pilot intervention by Liao et al. [16], the cross-sectional study by Byun et al. [17], and the large cohort by Bermingham et al. [18] were all assessed as having a low overall risk of bias. In contrast, the cross-sectional study by Sezer et al. [15] was deemed to have a serious overall risk of bias, primarily due to serious concerns regarding confounding factors that could have influenced the observed relationship between glycemic and blood pressure variability (Tables 3, 4).

**Table 3:** Risk of Bias Assessment for RCTs Using the Cochrane RoB 2 Tool This table reports the risk of bias assessment for the included RCTs (Zhang et al. [12] and Smith et al. [14]) using the Cochrane RoB 2 tool. Domains assessed included randomization process, deviations from intended interventions, missing outcome data, measurement of outcomes, and selection of reported results. Both studies were judged to have low risk of bias across all domains, indicating strong internal validity. RCT: randomized controlled trial; RoB 2: Risk of Bias 2

Study, year	D1: randomization process	D2: deviations from intended interventions	D3: missing outcome data	D4: measurement of the outcome	D5: selection of the reported result	Overall risk of bias
Zhang et al. [12] (2021)	Low	Low	Low	Low	Low	Low
Smith et al. [14] (2021)	Low	Low	Low	Low	Low	Low

**Table 4:** Risk of Bias Assessment for Non-randomized Studies Using the ROBINS-I Tool This table provides the methodological quality assessment of the non-randomized and observational studies [13,15-18] using the ROBINS-I tool. Risk of bias was evaluated across seven domains: confounding, participant selection, intervention classification, deviations from interventions, missing data, outcome measurement, and selection of reported results. Most studies were rated as low risk, except Sezer et al. [15], which was classified as having a serious risk of bias due to potential confounding. Overall, the non-randomized evidence was considered robust with minimal bias. ROBINS-I: Risk Of Bias In Non-randomized Studies of Interventions

Study, year	D1: confounding	D2: participant selection	D3: intervention classification	D4: deviations from interventions	D5: missing data	D6: outcome measurement	D7: selection of reported results	Overall risk of bias
Trim et al. [13] (2023)	Low	Low	Low	Low	Low	Low	Low	Low
Sezer et al. [15] (2021)	Serious	Low	Low	Moderate	Low	Low	Low	Serious
Liao et al. [16] (2020)	Low	Low	Low	Low	Low	Low	Low	Low
Byun et al. [17] (2020)	Low	Low	Low	Low	Low	Low	Low	Low
Bermingham et al. [18] (2022)	Low	Low	Low	Low	Low	Low	Low	Low

Discussion

This systematic review evaluated the emerging role of CGM in non-diabetic individuals as a tool for guiding lifestyle interventions aimed at cardiovascular prevention. By synthesizing evidence from seven heterogeneous studies, our findings indicate that CGM provides a powerful, real-time feedback mechanism that can personalize lifestyle modifications, particularly physical activity, leading to improved glycemic outcomes, which are established surrogates for cardiovascular risk. The most compelling evidence comes from intervention studies demonstrating that CGM can be used to tailor the timing of exercise, such as initiating walking based on an individual's PPGP, resulting in significant reductions in postprandial glycemia, insulin, and C-peptide [12]. This precise personalization moves beyond generic advice, leveraging CGM data to maximize the metabolic benefits of exercise. Furthermore, the use of CGM as a motivational tool, as seen in the pilot study by Liao et al. [16], highlights its potential to engage individuals in behavior change by providing tangible, immediate data on the physiological impact of their activities. The observational data further enrich our understanding, revealing that CGM can detect subtle dysregulation in states such as menopause [18] and OSA [17], identifying subpopulations that may be at a heightened cardiometabolic risk long before the onset of frank diabetes or CVD.

The efficacy of CGM-guided exercise timing, as demonstrated by Zhang et al. [12], aligns with and extends the principles of chrono-exercise, which posits that the timing of physical activity can influence its metabolic effects. Our review provides empirical support for this concept in a non-diabetic, at-risk population. The finding that activity timed before the glucose peak was more effective than activity at the peak underscores the value of CGM in moving from a reactive to a proactive management of metabolic health. This adds a layer of sophistication to existing public health guidelines, which generally recommend post-meal walking but do not specify individual peak timing. Similarly, the study by Smith et al. [14], which used CGM-prompted activity breaks to reduce fasting glucose and glycemic variability, reinforces the well-documented dangers of prolonged sitting. Our findings are consistent with a growing body of literature, such as the work by Dunstan et al. [19], which showed that breaking up sitting time with light-intensity walking improved glycemic control in overweight/obese adults, demonstrating that the beneficial effects of interrupting sedentariness are detectable and actionable through CGM-derived metrics even in the absence of diabetes.

The observed relationship between glycemic variability and cardiovascular risk surrogates, particularly blood pressure variability [15], is a critical finding of this review. This correlation in normotensive, normoglycemic individuals suggests that CGM-detected glycemic fluctuations may be an early marker of dysregulated autonomic function or endothelial dysfunction, both of which are precursors to hypertension and CVD. This echoes the findings of Campuzano et al. [20], who reported that increased glycemic variability, independent of mean glucose levels, was associated with adverse cardiovascular outcomes in high-risk populations. Our review suggests this relationship is present even in ostensibly healthy individuals, positioning CGM as a potential tool for early risk stratification. The data from Bermingham et al. [18] further strengthen this concept, linking the menopausal transition-a period of increased cardiovascular risk-to a deteriorative postprandial metabolic phenotype characterized by higher glucose excursions and variability, captured effectively by CGM. This is supported by previous work from Stancáková et al. [21], which identified postprandial metabolism as a key determinant of future diabetes and cardiovascular risk, highlighting the clinical relevance of the CGM metrics reported.

However, the direct evidence for CGM-guided interventions leading to improvements in traditional cardiovascular risk factors, such as blood pressure and lipids, remains limited within the current literature. Most included studies were either of short duration, focused on acute metabolic responses, or were observational in nature. For instance, while the intervention by Smith et al. [14] improved glycemic variability, it did not translate to significant changes in lipids or blood pressure over the three-week period. This is unsurprising, as changes in these harder endpoints typically require longer, more intensive interventions. This finding is consistent with the systematic review by Chehregosha et al. [22] on CGM in pre-diabetes, which found significant improvements in glycemic time-in-range but inconsistent effects on blood pressure and lipid profiles, suggesting that while CGM is excellent for guiding glucose-centric interventions, its impact on broader cardiovascular risk factors may be indirect or delayed. The primary cardiovascular benefit appears to be mediated through improvements in insulin sensitivity and beta-cell function, as indicated by the reductions in insulin and C-peptide in the study by Zhang et al. [12]. Improved insulin sensitivity is a cornerstone of cardiovascular health, as it is intimately linked to reduced inflammation, improved endothelial function, and favorable lipid metabolism, as extensively documented by Ormazabal et al. [23].

When considering the broader context of precision health, the use of CGM in non-diabetic individuals represents a paradigm shift toward proactive, personalized prevention. The ability of CGM to identify individual responses to dietary and activity challenges, as seen in the PREDICT study [18], moves beyond the one-size-fits-all approach. This aligns with the concepts explored by Zeevi et al. [24] in their landmark study on personalized nutrition, which demonstrated highly variable postprandial glycemic responses to identical foods, underscoring the limitation of generalized dietary advice. Our review suggests that CGM can operationalize this personalized approach for lifestyle intervention, empowering individuals to understand their unique metabolic responses and modify their behavior accordingly. The high acceptability and motivational impact reported by Liao et al. [16] are crucial for long-term adherence, a major challenge in lifestyle medicine. This is supported by the work by Allen et al. [25], which found that wearable devices providing real-time feedback can enhance engagement and sustain behavior change in weight management programs.

Nevertheless, it is important to contextualize the findings of the bed rest study by Trim et al. [13], which serves as a cautionary tale. Even with meticulous dietary adjustment to maintain energy balance, physical inactivity led to a profound deterioration in glucose homeostasis, including increased fasting insulin and reduced glucose disposal. This underscores that diet alone cannot counteract the detrimental metabolic effects of inactivity. The preservation of glycemic variability metrics in this controlled model, despite worsened insulin resistance, is intriguing and may suggest that these metrics are more sensitive to dietary composition and meal timing than to the state of physical inactivity under energy-balanced conditions. This finding partially contrasts with epidemiological studies like that by Hamburg et al. [26], which associated sedentary behavior with adverse metabolic profiles, but highlights the complex interplay between energy intake, expenditure, and macronutrient balance that CGM can help elucidate.

Limitations

This systematic review has several limitations that must be acknowledged. First, the number of studies meeting the inclusion criteria was small (n = 7), and they were highly heterogeneous in terms of design, population, intervention, and outcomes, precluding a meta-analysis and limiting the strength of our conclusions. Second, the duration of the interventions was generally short, ranging from a single night to several weeks, which is insufficient to assess the long-term sustainability of CGM-guided interventions or their impact on hard cardiovascular endpoints. Third, the majority of evidence revolves around glycemic outcomes, with direct measures of cardiovascular health (e.g., blood pressure, lipids, and arterial stiffness) being less frequently reported. Fourth, the risk of bias was serious in one observational study due to confounding [15], and while the others were low risk, the non-randomized nature of most studies limits causal inference. Furthermore, the populations studied were often specific (e.g., overweight men and post-menopausal women), which may affect the generalizability of the findings to the broader non-diabetic population. Finally, the cost, accessibility, and interpretation of CGM data present significant practical barriers to its widespread implementation for primary cardiovascular prevention at the current time.

## Conclusions

This systematic review provides preliminary but promising evidence that CGM can be a valuable tool for guiding personalized lifestyle interventions in non-diabetic individuals for cardiovascular prevention. CGM enables the precise timing of physical activity, serves as a potent motivational feedback mechanism, and identifies at-risk metabolic phenotypes long before traditional diagnostic thresholds are crossed. The primary benefits observed are improvements in glycemic parameters, particularly postprandial glucose and glycemic variability, which are well-established surrogate markers for insulin resistance and future cardiovascular risk. While direct evidence of impact on classic cardiovascular risk factors is still nascent, the improvement in insulin sensitivity mediated by CGM-guided interventions is a fundamentally important cardiometabolic benefit. CGM could complement existing cardiovascular prevention frameworks by providing real-time, individualized metabolic feedback that enhances the effectiveness of conventional lifestyle and risk factor management strategies. Future research should prioritize long-term, large-scale RCTs that investigate the sustained effect of CGM-guided lifestyle modifications on comprehensive cardiovascular risk profiles, including imaging and biomarker endpoints, in diverse non-diabetic populations. Overcoming the practical and economic barriers to CGM use will be essential for translating this promising technology into effective public health strategies for primary cardiovascular prevention.
